# REST is a hypoxia-responsive transcriptional repressor

**DOI:** 10.1038/srep31355

**Published:** 2016-08-17

**Authors:** Miguel A. S. Cavadas, Marion Mesnieres, Bianca Crifo, Mario C. Manresa, Andrew C. Selfridge, Ciara E. Keogh, Zsolt Fabian, Carsten C. Scholz, Karen A. Nolan, Liliane M. A. Rocha, Murtaza M. Tambuwala, Stuart Brown, Anita Wdowicz, Danielle Corbett, Keith J. Murphy, Catherine Godson, Eoin P. Cummins, Cormac T. Taylor, Alex Cheong

**Affiliations:** 1 Systems Biology Ireland, University College Dublin, Dublin 4, Ireland; 2 Conway Institute of Biomolecular and Biomedical Research, School of Medicine and Medical Sciences, University College Dublin, Dublin 4, Ireland; 3 Instituto Gulbenkian de Ciência, Rua da Quinta Grande, 2780-156 Oeiras, Portugal; 4 Institute of Physiology and Zurich Centre for Integrative Human Physiology, University of Zurich, Zurich, Switzerland; 5 Diabetes Complications Research Centre, School of Medicine and Medical Sciences, University College Dublin, Dublin 4, Ireland; 6 Faculdade de Medicina, Universidade de Lisboa, 1649-028 Lisbon, Portugal; 7 School of Pharmacy and Pharmaceutical Sciences, University of Ulster, Coleraine, Co. Londonderry, BT52 1SA, Northern Ireland, UK; 8 Center for Health Informatics and Bioinformatics, New York University School of Medicine, New York, NY 10016, USA; 9 Neurotherapeutics Research Group, UCD School of Biomolecular and Biomedical Science, Conway Institute, University College Dublin, Belfield, Dublin 4, Ireland; 10 Life and Health Sciences, Aston University, Birmingham, B4 7ET, UK

## Abstract

Cellular exposure to hypoxia results in altered gene expression in a range of physiologic and pathophysiologic states. Discrete cohorts of genes can be either up- or down-regulated in response to hypoxia. While the Hypoxia-Inducible Factor (HIF) is the primary driver of hypoxia-induced adaptive gene expression, less is known about the signalling mechanisms regulating hypoxia-dependent gene repression. Using RNA-seq, we demonstrate that equivalent numbers of genes are induced and repressed in human embryonic kidney (HEK293) cells. We demonstrate that nuclear localization of the Repressor Element 1-Silencing Transcription factor (REST) is induced in hypoxia and that REST is responsible for regulating approximately 20% of the hypoxia-repressed genes. Using chromatin immunoprecipitation assays we demonstrate that REST-dependent gene repression is at least in part mediated by direct binding to the promoters of target genes. Based on these data, we propose that REST is a key mediator of gene repression in hypoxia.

Hypoxia is a feature of a range of physiological and pathophysiological conditions including embryonic development, exercise, cancer, ischemia and inflammation^1^. Throughout evolution, adaptive pathways have developed to help an organism cope with hypoxia. The best-described transcriptional adaptive response in cells is mediated by the hypoxia inducible factor (HIF) signalling pathway, which up-regulates genes which restore oxygen and energy homeostasis^2^,^3^,^4^. In normoxia, HIFα is hydroxylated by the prolyl-hydroxylase domain (PHD) family of dioxygenases targeting it for ubiquitylation by the von Hipple Lindau E3 ligase complex and subsequent proteosomal degradation^5^. This process is reversed in hypoxia and HIFα is stabilized, dimerises with HIFβ and binds to hypoxia response elements (HRE) in the regulatory regions of target genes^6^. HIF drives an adaptive response to hypoxia by promoting the expression of genes that regulate erythropoiesis, angiogenesis and glycolysis^6^. However in cancer, HIF signalling can be maladaptive and contribute to tumour survival^7^.

Microarray studies of mammalian cells exposed to hypoxia have shown that approximately the same numbers of genes are repressed and induced^2^,^3^,^8^. While HIF has been described as a master regulator of gene expression in hypoxia, a significant number of other transcription factors are also hypoxia-sensitive and control HIF-independent gene expression^9^. Microarray data combined with siRNA against HIF-1/-2 indicate that there are HIF-dependent and HIF-independent genes which are differentially expressed in hypoxia^2^. ChIP-seq and microarray data indicate that while HIF is enriched in the promoters of genes induced in a HIF-dependent way, it is not enriched in the promoters of genes that are repressed in hypoxia, thus indicating the presence of HIF-independent and/or indirectly HIF-dependent mechanisms governing gene repression in hypoxia^10^. While HIF is well known to regulate gene induction^2^,^3^,^4^, the mechanisms underpinning transcriptional repression of genes in hypoxia remain poorly understood and is the topic of the current study^8^,^11^. Transcriptional repressors are a large and diverse group of proteins^12^. Mechanistically repressors can act by inhibiting the basal transcription machinery, ablation of activator function and remodelling of chromatin. They can be further classified into DNA-binding proteins (Class I) like Krüppel zinc fingers, proteins that bind DNA-binding proteins (Class II) such as the DNA-methyltransferase Dnmt3, or proteins that target activators, reducing their activity, such as IκB that sequesters NFκB in the cytosol^12^.

The Repressor Element 1-Silencing Transcription Factor (REST) is a C2H2- or Krüppel-type zinc finger, one of the largest classes of transcription factors in humans^13^. It binds to the 21 base pair Repressor Element 1 (RE1) on the promoter of target genes and inhibits transcription by regulating chromatin structure or by inhibiting the basal transcriptional machinery^14^. Proteosomal REST degradation is induced during neuronal differentiation, resulting in the promotion of the expression of the genes which confer the unique neuronal phenotype^14^,^15^. Thus, REST was initially regarded as a master regulator of neurogenesis and as the first example of a vertebrate transcription silencer protein that regulates a large repertoire of cell type-specific genes^16^. However, REST has since being shown to be implicated in the regulation of non-neuronal biological processes in a variety of cell types, including cardiac myocytes, immune, vascular and tumour cells^15^,^17^,^18^,^19^. In the aging human brain, REST is induced to protect against oxidative stress and brain neurodegeneration^20^. Of particular interest for our study, ischemia and/or oxygen-glucose deprivation have previously been shown to induce REST nuclear protein and mRNA^21^,^22^ and to modulate the expression of target genes^21^. Furthermore, we have recently shown that REST represses the HIF-1α mRNA expression and contributes to the resolution of the HIF response during prolonged hypoxia^23^. This led us to explore the global response of REST to cellular hypoxia, with the aim of developing our understanding of the signalling mechanisms underpinning hypoxia-dependent transcriptional repression.

Using an unbiased, high-throughput approach combined with biochemical analysis, we demonstrate that REST accumulates in the nucleus of cells exposed to hypoxia and acts as a key repressor of the hypoxic transcriptome, regulating approximately 20% of the hypoxia-repressed genes. Furthermore, hypoxia leads to a change in the target gene repertoire of REST, from the repression of neuronal genes in normoxic/basal conditions, to the repression of metabolic, cell cycle and proliferation related genes in hypoxia. Together these findings indicate a previously unknown key role for the tumour suppressor REST in hypoxic signalling.

## Results

### High-throughput analysis of the transcriptional responses to hypoxia

**Figure 1:**
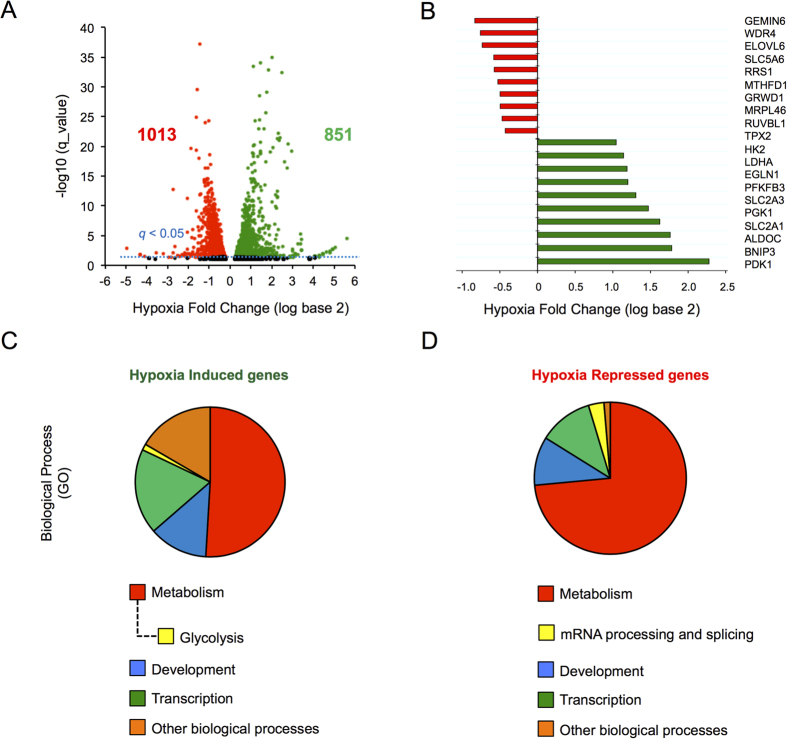
Hypoxia induced gene expression changes assessed by genome wide sequencing. (**A**) Volcano plot showing changes in gene expression due to hypoxia (1% O_2_) plotted against significance. Each dot represents the mean fold change for a single gene with induced genes as green dots, repressed as red and unchanged as black. Horizontal blue dashed line indicates *q *< 0.05. Cells were treated with Ctrl_RNAi before hypoxia exposure and RNA-seq. (**B**) Mean fold change values for well described hypoxia induced (green bars) and repressed genes (red bars). (**C**,**D)** Enriched GO Biological processes.

Previous microarray-based transcriptomic analysis of changes in global mRNA expression in response to hypoxia revealed that down-regulated genes reach their maximal repression following prolonged hypoxic exposure, while induced transcripts generally reach their maximum induction at earlier time-points^8^. Here we used human embryonic kidney (HEK293) cells exposed to normoxia or hypoxia for 24 hours as a cell model to investigate the processes associated with gene repression. High-throughput sequencing of poly(A)+ RNA (RNA-Seq) was performed on samples collected and transcript analysis revealed the presence of almost 2000 genes that were differentially expressed in hypoxia, with similar numbers of genes being induced (green; 851) and repressed (red; 1013) (Fig. 1A). These data are quantitatively consistent with previously published transcriptomic studies of human cells exposed to hypoxia^2^,^3^,^8^. Importantly, cohorts of well-characterized hypoxia-induced genes were up regulated including *LDHA, HK2, SLC2A1*, *EGLN1* and *BNIP3* (Fig. 1B). Furthermore, a number of previously characterized hypoxia-repressed genes were present in the down-regulated cohort including *RRS1* (involved in ribosome biogenesis) and *MTHFD1* (involved in *de novo* purine synthesis) (Fig. 1B)^3^,^8^. Therefore, hypoxia had comparable effects on global gene induction and repression in HEK293 cells.

Using PANTHER ontological analysis, we classified hypoxia-induced and repressed genes according to gene ontology (http://www.pantherdb.org/). Our analysis revealed that hypoxia-induced and repressed genes could be associated with either similar or distinct processes (Figs 1C,D and S1). For example, the glycolytic pathway, known previously to be induced in response to hypoxia^1^, was identified only on the list of hypoxia-induced metabolic pathways (Figs 1C and S1G) while genes encoding proteins associated with intercellular junctions were identified only in the hypoxia-repressed gene cohort (Figure S1F). Although both induced and repressed gene cohorts included genes involved in the regulation of transcription, metabolism and development, only the repressed cohort contained an overrepresentation of genes involved in mRNA processing and splicing (Fig. 1D). Taken together, these results indicate that hypoxia alters the expression of an equivalent number of increased and decreased genes which have both common and distinct roles in regulating cell responses.

### REST is a hypoxia sensitive transcription factor

**Figure 2:**
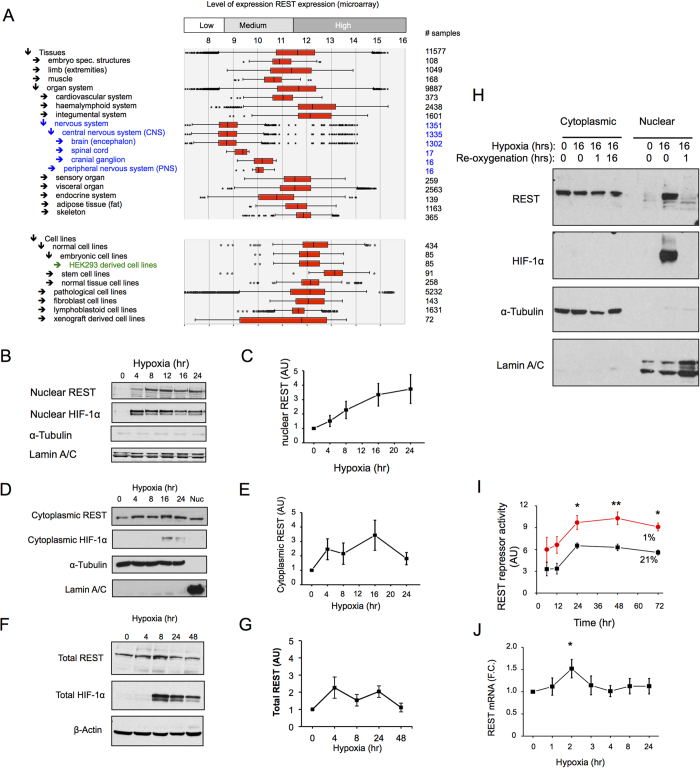
REST is a hypoxia sensitive transcription factor. (**A)** REST mRNA expression compiled from publically available microarray datasets of different tissues and cell lines. The graph was compiled with Genevestigator and shows the human REST mRNA expression levels (xx axis) and number of samples (yy). HEK293 cells were exposed to hypoxia (1% O_2_) for the indicated time points, nuclear extracts **(B)**, cytoplasmic **(D)** and whole cell extracts **(F)** were prepared and immunoblotted as indicated for REST, HIF-1α, Lamin A/C (nuclear marker) and α-Tubulin (cytoplasmic marker) (n = 4–5). (**C,E,G**) Densitometric analysis of B, D and F, respectively. **(H)** HEK293 cells where exposed to hypoxia (1% O_2_) for 16 hours followed by re-oxygenation (21% O_2_) for 1 and 16 hours, cytoplasmic and nuclear extracts where prepared and immunoblotted as indicated (N = 4). (**I**) Cells were transfected with a vector expressing *Gaussia* luciferase under the control of the TK promoter (pTK-GLuc) for constitutive expression, or with the REST responsive pRE1-TK-GLuc construct. REST repressor activity = RLU _pTK-GLuc_ / RLU _pRE1-TK-GLuc_ (N = 3). (**J**) HEK293 cells were exposed to hypoxia (1% O_2_) for the indicated time points, mRNA was collected and analysed by qRT-PCR, n = 6–10. Data are represented as mean ± SEM. *p < 0.05, **p < 0.01, significant fold change over 21% O_2_.

We next focused on possible molecular mechanisms underpinning the repression of gene transcription in hypoxia. REST was initially identified as a regulator of neuronal gene expression, but was subsequently shown to have non-neuronal roles^17^,^18^,^19^. Using publically available microarray datasets, we confirm that REST is extensively expressed in multiple tissue types and cell lines, being highly expressed in HEK293 cells used in this study (Figs 2A and S2). HEK293 cells have been instrumental for the discovery of various aspects of REST biology: transcriptional networks^24^, phosphorylation and the proteasome system^25^ and regulation of HIF-1α in hypoxia^23^. Similar levels of REST could be detected in the nucleus and cytoplasm of normoxic HEK293 cells (Figure S3). In response to hypoxia, there was a more pronounced increase in REST levels in the nuclear (Fig. 2B,C) than in either the cytoplasmic (Fig. 2D,E) or whole cell extracts (Fig. 2F,G), suggesting nuclear accumulation as the major response of REST to hypoxia. This nuclear accumulation was also observed in MCF10A (Figure S4). We investigated the effect of re-oxygenation on nuclear REST levels, and found that accumulation in hypoxia was reversible within one hour of re-oxygenation (Fig. 2H), strongly supporting an oxygen-regulated post-translational control mechanism of REST cellular localization from the cytoplasm to the nucleus and back.

The increase in nuclear REST protein was associated with an increase in REST-dependent repressor activity, as measured using by a repressor element-1 (RE1)-luciferase assay developed to measure REST transcriptional activity (Figs 2I and S5). REST mRNA expression during hypoxic exposure was modestly and transiently increased, and was statistically significant only at the 2 hours time point (Fig. 2J).

To assess the effect of hypoxia on REST protein stability, HEK293 cells were exposed to 24 hours of either normoxia (21% oxygen) or hypoxia (1% oxygen), followed by cycloheximide treatment in normoxia and protein stability was monitored by western blot. REST displayed a long half-life that was not increased by hypoxia (Figures S6A,B). In contrast, HIF-1α in the same cells exhibited a short half-life, with hypoxia-stabilized HIF-1α being completely degraded within 1 hour of culture in normoxia (Figure S6C). β-Actin exhibited a long half-life that was also not affected by hypoxia (Figure S6D).

Taken together, these results suggest that hypoxia leads to REST nuclear translocation, through a post-translational oxygen regulated mechanism, with a modest increase in transcription and, unlike HIF, independent of altered protein stability.

### Role of REST in basal gene expression in hypoxia

**Figure 3:**
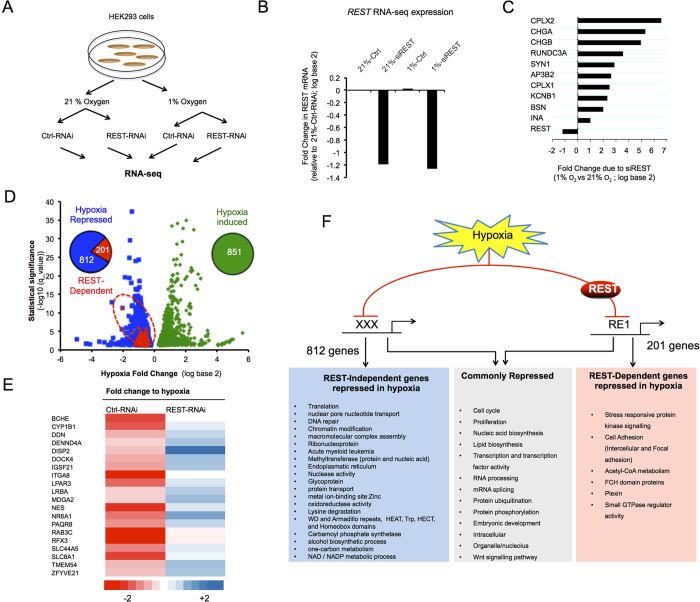
REST regulates hypoxia repressed genes. (**A)** Overview of the RNA-seq approach used to reveal the contribution of REST to gene repression in hypoxia. **(B)** REST mRNA expression from the 3 independent RNA-seq experiments after treatment with hypoxia or REST-RNAi treatment. **(C)** REST-RNAi treatment on cells exposed to normoxia (21% oxygen) for 24 hours led to a significant increase in the expression of well-characterised REST target genes (shown as Cuffdiff mean fold changes (log base 2) of 3 independent experiments). **(D)** RNA-Seq analysis revealed 201 REST-Dependent genes (Red) among the repressed genes (Blue). Data shown as a Volcano plot showing changes in gene expression due to hypoxia (1% O_2_) plotted against significance. Only genes with statistically significant fold changes are shown. Each dot represents the mean fold change for a single gene with induced-genes as green dots (“Hypoxia induced”), repressed-genes as blue (“Hypoxia repressed”), and the genes that were not repressed by hypoxia in the presence of siRNA against REST shown as red dots (“REST-Dependent”). **(E)** Heat-map showing genes repressed by hypoxia (left column, Ctrl-RNAi), that where not repressed upon REST-RNAi treatment (right column, REST-RNAi). Data is presented as fold change to hypoxia of the top 20 genes most repressed by hypoxia among the REST-dependent genes. **(F)** Summary of the gene clusters found to be uniquely repressed by REST-Independent mechanisms (Blue), REST-Dependent mechanisms (Red) and the common clusters between the two mechanisms (Grey). Clusters were obtained using DAVID Functional Annotation Clustering together with manual curation of the results. For a detailed description of the annotation terms and gene groups associated with each cluster see Table S2.

The observation that nuclear REST expression and activity are increased led us to hypothesize that REST may play a broad role in regulating gene repression in hypoxia. To address this, we performed RNA-Seq analysis of hypoxic cells in the presence of scrambled or REST-specific siRNA (Fig. 3A). An overview of the different steps performed to identify differentially expressed genes is shown in Figure S7A. All quality control checks are shown in Figures S7B–E and S8. As described earlier (Fig. 1B), a cohort of known hypoxia-inducible genes served as positive experimental controls. REST-RNAi treatment led to a decrease in REST mRNA (Figs 3B and S9A) and protein expression (Fig. S9B,C) and a resultant increase in a number of previously identified REST target genes^24^,^26^ in 21% oxygen (Fig. 3C), thus validating the effectiveness of the REST-RNAi knockdown.

Among the genes whose basal expression was reduced in hypoxia (1013 genes represented by blue and red dots in Fig. 3D), 201 (~20%) were found to be REST-Dependent (red dots in Fig. 3D), i.e. they were not significantly repressed by hypoxia when REST was silenced (Fig. 3E and Supplemental Table S1). On the other hand, 812 genes repressed in hypoxia were found to be REST-Independent, suggesting alternative gene regulation (Supplemental Table S1). An ontological and clustering analysis of the hypoxia-repressed genes showed that metabolic processes and other energy demanding processes including transcription, proliferation and cell cycle were heavily over-represented in the cohort repressed in a hypoxia and REST-Dependent manner (Fig. 3F and Supplemental Table S2). Analysis of cell death in hypoxic cells treated with RNAi targeting REST did not reveal any effect of REST on cell death (Figure S10). Altogether, these data indicate that REST is an important transcriptional repressor in hypoxia, whose major function is beyond the control of cell survival in hypoxia.

### REST regulates specific gene cohorts in normoxia and hypoxia by direct binding to gene promoters

**Figure 4:**
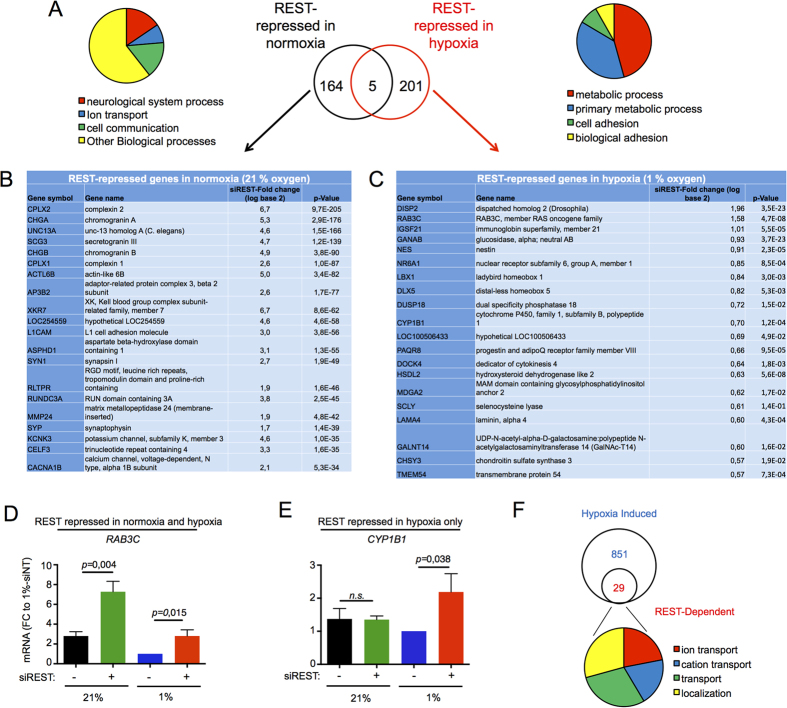
REST regulates different transcriptional networks in normoxia and hypoxia. (**A**) There is limited overlap (5 genes) between REST-Dependent genes repressed in normoxia (164, dark circle) and REST-Dependent genes repressed in hypoxia (201, red circle). Circular diagrams show the distribution of enriched GO biological processes by PANTHER overrepresentation test. Significance was set at p < 0.05. The top 20 genes by fold-change upon REST-RNAi treatment for the REST-Dependent genes repressed in normoxia **(B)** and REST-dependent genes repressed in hypoxia **(C)** are shown. **(D,E)** qRT-PCR was used to validate the mRNA expression of the REST target genes *RAB3C* and *CYP1B1* in cells exposed for 24 hours to normoxia (21% oxygen) or hypoxia (1% oxygen), in the presence or absence of an siRNA targeting REST. **(F)** Of the 851 genes induced in hypoxia (blue circle), 29 were further induced by REST siRNA treatment. Circular diagrams show the distribution of enriched GO biological processes by PANTHER overrepresentation test.

Comparison of the REST-dependent genes repressed in hypoxia to the REST-Dependent genes repressed in normoxia revealed a limited overlap (5 genes, Fig. 4A and Supplemental Table S3). Ontological analysis revealed well characterised neuronal genes regulated by REST in normoxia (Fig. 4A black circle and 4B). A different subset of genes were found for REST-repressed genes in hypoxia (Fig. 4A red circle and 4C), with ontological analysis showing an enrichment for metabolic and cell adhesion processes (Fig. 4A). Among the 10 genes most sensitive to REST regulation in hypoxia (Fig. 4C), *RAB3C* (encoding for RAB3C GTPase), is predicted to be regulated by REST in normoxia and hypoxia, while *CYP1B1* (encoding for cytochrome P450 1B1) is predicted to be regulated only in hypoxia. Using qRT-PCR we tested and validated these findings (Fig. 4D,E). 29 genes were further induced by REST siRNA treatment (Fig. 4F and Supplementary Table S5). Together, these results indicate that hypoxia leads to the recruitment of REST to a largely different set of genes from those regulated by REST in normoxia. We tested a further group of 6 genes for their regulation by REST in hypoxia. While we observed a similar trend in the rescue of repression by siREST, only 1 gene, *GANAB* (encoding for the neutral alpha-glucosidase AB in the glycan metabolism pathway) was significantly de-repressed (Figure S11). Overall, we confirmed that 3 out of 8 genes were bona fide REST target genes in hypoxia, from which so we could assume a similar percentage of the 201 newly identified REST target genes in hypoxia to be similarly genuine (38%, about 75 genes).

**Figure 5:**
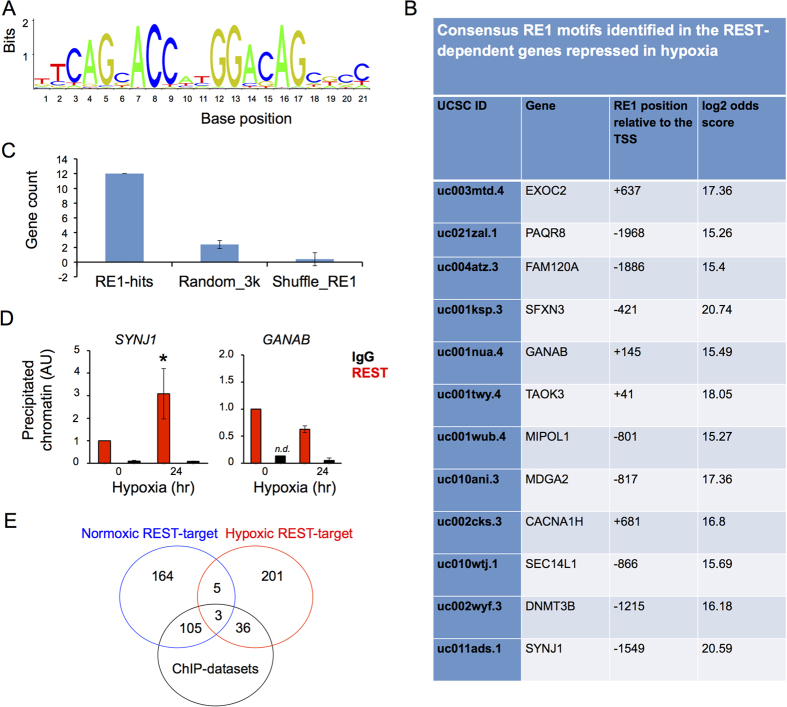
REST represses genes in hypoxia by direct binding to an RE1 site on their promoter regions. (**A**) Consensus RE1 binding site (JASPAR matrix representation). (**B**) Identification of 12 high stringency RE1 sites in the in the proximal promoter (−2000 bp to +1000 bp kilobase) of REST-dependent hypoxia repressed genes. (**C**) The number of RE1 sites identified by our informatics analysis is compared to the number of RE1 sites that could be expected by random chance (see Materials and Methods for details. Data is presented as mean ± SD. Error bars represent the SD from the 500 simulations. (**D**) ChIP assay for REST binding in normoxia and hypoxia (24 hours) to the RE1 found in the proximal promoter of the *GANAB* and *SYNJ1* gene. (**E**) Overlap between the number of genes identified to be REST target genes in normoxia or hypoxia, with the REST target genes identified by ChIP-seq in published datasets. Data is represented as mean ± SEM. *p < 0.05, significant over normoxic control and IgG. *n.d.* = none detected, for some of the biological replicates.

In order to test if the REST regulation of hypoxia repressed gene is direct or indirect, we searched for the consensus RE1 motif (Fig. 5A) in the region from −2 kilobase pairs to +1 kilobase pairs from the transcription start site in the cohort of genes repressed by REST in hypoxia. In this search, we found high stringency consensus RE1 sites in just 12 genes (Fig. 5B,C). This indicates that, in the majority of cases, REST is binding to distant or non-consensus sites. ChIP assays were performed to validate REST binding to the RE1 site on the promoters of *SYNJ1* (encoding for synaptojanin 1) and *GANAB*, two of the identified REST target genes in hypoxia (Fig. 5B,D). For both *SYNJ1* and *GANAB*, we detected REST binding to their promoters in hypoxia (Fig. 5D). For SYNJ1, we could observe a significant recruitment of REST in hypoxia over normoxia. We have also investigated whether the hypoxic REST-repressed genes from our RNA-seq are similar to published REST ChIP-Seq datasets from 4 different cell lines^27^,^28^,^29^. We found that, out of our list of 201 genes identified as REST-repressed in hypoxia, 36 were also REST target genes from the published ChIP-sequencing experiments (Fig. 5E and Supplementary Table 4). This probably is still an under-estimation as there are no publically available REST ChIP-seq data performed on hypoxic cells.

**Figure 6:**
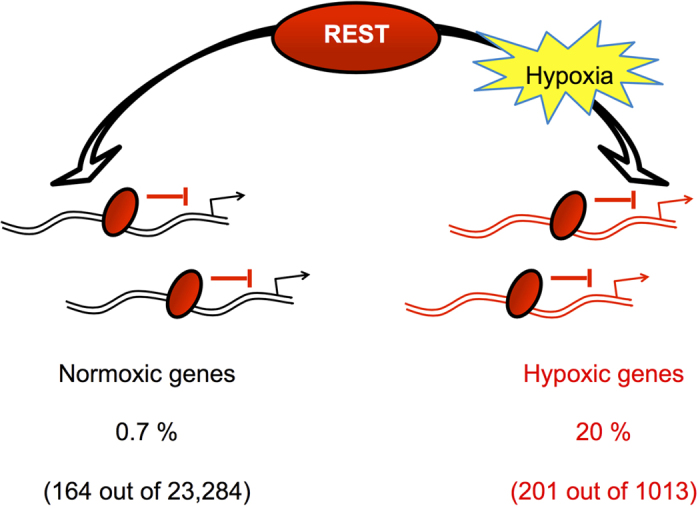
REST is a master regulator of gene repression in hypoxia and regulates different transcriptional networks depending on oxygen availability. Our analysis showed that REST repressed 20% of the hypoxia-repressed genes, and only 0.7% of all genes repressed in normoxia. Furthermore there is only a limited overlap between the 2 transcriptional networks (5 genes), and most of the genes repressed by REST in normoxia are still repressed by REST under hypoxia. Thus, rather than a shift on the transcriptional repertoire of REST target genes from normoxia to hypoxia, we show that there is a recruitment to new target genes in hypoxia.

Thus our unbiased approach using RNA-Seq revealed a previously unknown aspect of REST repression and identifies an extensive repertoire of genes regulated by REST in hypoxia (Fig. 6), providing new insight into the mechanisms of gene repression in hypoxia.

## Discussion

While HIF is considered to be the master regulator of increased gene expression in hypoxia^2^,^3^,^4^, the mechanisms of transcriptional repression in hypoxia are poorly understood^11^. Our current study identifies REST as a key negative regulator of gene expression in hypoxia. We show it accumulates in the nucleus in response to hypoxia, and is responsible for the repression of approximately 20% of genes downregulated in hypoxia, thus acting as a counter-regulator to HIF-dependent gene expression.

REST is a phosphoprotein whose protein stability is tightly regulated during development. REST levels are reduced during neuronal development to allow the expression of REST target genes, while outside the nervous system, REST levels remain high to repress neuronal specific genes outside the nervous systems^14^. REST protein was stabilised in an *in vitro* model of ischemia (typically 10 to 30 minutes of glucose/oxygen deprivation followed by re-oxygenation, typically for 24 hours) due to an inhibition of its degradation^30^. Our pulse-chase experiments with cycloheximide showed that hypoxia did not affect the stability of REST. Instead, there was increased nuclear localisation. We also noticed that nuclear REST seemed to run at a smaller size. As REST is known to be phosphorylated and ubiquitylated^25^, it is possible that post-translational modifications also control its translocation to the nucleus.

Our experiments using REST siRNA in normoxic cells show that, in the basal state, only 164 genes of the detected normoxic transcriptome (23,284 genes) are repressed by REST (equivalent to approximately 0.7% of the total transcriptome detected). Of the total of genes repressed in hypoxia (1013), we found that 201 gene are significantly repressed by REST in hypoxia (equivalent to approximately 20% of the total), placing REST as a significant repressor of gene expression in hypoxia (Fig. 6). Using qRT-PCR, we had a 38% validation rate, suggesting about 75 of the 201 genes are expected to be *bona fide* REST targets in hypoxia. Furthermore, we found a small overlap (5 genes) between the REST-dependent genes in normoxia compared with the REST-dependent genes in hypoxia, indicating that REST regulates context-specific transcriptional networks. This has been previously described in other systems: for example REST was shown to regulate different target genes in neuronal stem cells versus embryonic stem cells^31^, embryonic stem cells vs epiblast stem cells^32^ and in response to ischemia in hippocampal neurons^21^. The factors leading to this differential recruitment are only starting to be uncovered, and may involve different co-factors, e.g. mSin3A^32^ and differences in the REST binding sequences^31^,^33^. When we compared our normoxic/hypoxic REST target genes dataset with published ChIP-seq datasets, we found similarities of about 64% with the normoxic genes and 18% with the hypoxic genes. This difference in proportions could be due to REST in hypoxia regulating more genes indirectly than by direct binding. This simple analysis reveals that there are layers of regulations in the control of gene expression and could open new research avenues into direct and indirect REST regulation.

Our ChIP and qRT-PCR experiments reveal two distinct binding patterns in 2 identified REST target genes *SYNJ1* and *GANAB*. While REST was bound to both of their promoters in normoxia, we observed significant increased binding of REST to only the *SYNJ1* promoter in hypoxia. We propose that, while REST recruitment is correlated to gene repression in hypoxia for some REST target genes (e.g. *SYNJ1*), for other genes (e.g. *GANAB*) REST might be already bound to the target gene in a low affinity state, “poised to act,” with its repressive activity triggered only in hypoxia, possibly by co-factor recruitment. This is similar to the findings of Otto and colleagues^34^, which show that many of their identified REST target genes were still expressed while others were repressed, potentially due to their functional relevance to the cell. Interestingly *SYNJ1* encodes for synaptojanin, a protein involved in clathrin-mediated endocytosis. It could be speculated that this ATP-intensive process is marked for repression as soon as cellular levels of ATP become scarce.

Our RNA-seq experiment was performed on cells in hypoxia for 24 hours (to maximise gene repression^8^) and we did not observe as dramatic an increase in HIF and its target genes as what we have previously reported in cells exposed to 8 hours of hypoxia^23^. Thus our experiments demonstrate the temporal nature of HIF signalling and its tight regulation by multiple transcription factors such as REST, NFκB^23^,^35^ (which promotes *HIF1A* mRNA expression), or miR-155^36^ (which de-stabilizes *HIF1A*). Of note, our previous study showed that NFkB is recruited to the *HIF1A* promoter with maximal binding at 8 hours in hypoxia and back to normoxic level after 24 hours^23^. It is thus possible that in the absence of REST (the repressor), we see increased *HIF1A* mRNA at 8 hours, but not at 24 hours, because one of the activators NFκB is less bound, or less active at this particular time point. The HIF signalling network is ripe for mathematical modelling to unravel its regulatory pathways^37^.

Gene ontology analysis suggests that REST plays an important role in the cellular adaptation to hypoxia by supressing genes involved in proliferation (*DLX5, PRKCB* and *MET*), cell cycle progression (*TBX3, CABLES1, ARID3A* and *ARAP1*) and transcription, with transcription factors (TF) (*TCF12* and *LBX1*), regulators of TF activity (*MYCBP* and *ATF7IP*) and general transcription apparatus (*MMS19, CDK19* and *MED12*), consistent with our hypothesis that direct and indirect mechanisms contribute to REST gene repression in hypoxia. In addition REST also seems to regulate important metabolic processes including the biosynthesis of lipids (*MBOAT2, GPAM, PIGN*, *AGPS* and *ACACA*) and nucleic acids (*PARP4, SKIV2L2, PLRG1 and DHX37*). Interestingly REST also represses genes involved in protein catabolism (*FBXO18, NEDD4*, *ERLIN2* and *TRIP12*). Our own ChIP experiments confirmed the hypoxic regulation of SYJN1 encoding synaptojanin 1, a protein involved in clathrin-mediated endocytosis. Thus repressing ATP-intensive processes in hypoxia is a plausible function for REST. Our lab has also shown a role for REST in the regulation of HIF-1α, and thus could impact on HIF-regulated genes as well^23^. Thus our transcriptomic analysis of hypoxic cells indicates that REST might play a key role in the regulation of proliferation and metabolic processes in hypoxia.

Some of the REST functions may be maladaptive in cancer. For example, REST-Dependent repression of cell adhesion genes in hypoxia (*ICAM5, ITGA8, SORBS1, LAMA4, LAMC1, LAMB1, COL14A1* and *COL4A5*) might be of importance for metastasis as hypoxic suppression of adhesion molecules in cancer cells has been proposed as a mechanism to allow hypoxic cancer cells to escape their stressed environment^38^,^39^. Some of the previously described genes are part of cancer pathways (*RB1, MET, MYCBP, WNT5A, HDAC1, PRKCB, LAM4A, LAMC1 and LAMB1*). The significance of the hypoxic repression of these genes by REST will be context- and cell-specific as REST may play both tumour suppressor and oncogenic roles, a feature it shares with NOTCH signalling^40^, E-cadherin^41^ and MYC^42^. Indeed, a recent study^43^ showed that knockdown of REST in normoxic prostate cancer cells can induce HIF signalling pathway, itself also implicated in cancer. In neuronal cancer, REST expression is high and has oncogenic properties by being anti-apoptotic and pro-tumorigenic^15^,^44^,^45^. We speculate that the hypoxic microenvironment present in these tumours^46^ could be a potential mechanism explaining REST over-expression. This is further supported by the observation that hypoxic neuroblastoma tumours and cells exposed to hypoxia (1% oxygen) down-regulate neuronal markers^47^,^48^. REST is also implicated in stem cell renewal, and has been recently reported to accumulate in the nucleus of Marrow-isolated multilineage inducible (MIAMI) cells exposed to 3% oxygen^49^.

Despite being widely regarded as an activator of gene expression in hypoxia, there are a few genes reported to be directly repressed by HIF-1α in hypoxia (e.g. *CFTR*^50^, *ADK*^51^ and *APC*^52^). Other examples of hypoxia-induced transcriptional repressors include DEC1 (differentially expressed in chondrocytes protein 1) which represses MITF (Microphthalmia-associated transcription factor)^53^ and Bach1 (BTB and CNC homology 1, basic leucine zipper transcription factor 1) which represses HO-1 (heme oxygenase-1)^54^. However, none of these appear to regulate as broad a range of genes in hypoxia as REST. Indeed, our data indicates that REST regulates 20% of genes repressed in hypoxia, which may be a key and previously unappreciated feature of the hypoxic response. REST-repressed genes regulate biosynthetic metabolism, cell cycle and proliferation indicating a major role in the cellular adaptation to hypoxia. The whole set of 201 genes repressed in hypoxia holds exciting possibilities regarding the discovery of novel mechanisms in the hypoxic response mediated by transcriptional repression. In summary, our findings have identified REST as a key regulator underlying gene repression and cellular adaptation in hypoxia.

## Methods

### Cell culture

Human embryonic kidney cells (HEK-293) were cultured in Dulbecco’s modified eagle medium (DMEM, high glucose 4.5 g/L without pyruvate) supplemented with 10% foetal calf serum (FCS) and 100 U/mL penicillin-streptomycin (PS). Human non-tumorigenic mammary epithelial cells (MCF10A) were grown in DMEM/F12 with 5% horse serum, 100 U/mL PS, 2 mM L-Glutamine, 10 μg/mL insulin, 20 ng/mL Epidermal Growth Factor (EGF), 0,5 μg/mL hydrocortisone and 100 ng/ml cholera toxin. All cells were obtained from the American Type Culture Collection (ATCC). All reagents for cell culture were from Gibco (Life Technologies, Calrsbad, CA, USA), unless otherwise stated. Cells were exposed to hypoxia using pre-equilibrated media and maintained in standard normobaric hypoxic conditions (1% O_2_, 5% CO_2_ and 94% N_2_) in a hypoxia chamber (Coy Laboratories, Grass Lake, Michigan, USA). Normoxic controls were maintained at atmospheric O_2_ levels (21% O_2_, 5% CO_2_ and 74% N_2_) in a tissue culture incubator. Re-oxygenation was performed by bringing hypoxic cells to the tissue culture incubator for the indicated time points.

### Cloning

All reagents were from New England Biolabs (NEB) unless otherwise stated. The pRE1-TK-GLuc REST responsive construct was developed by incorporating the RE1 of the *SCN2A* gene^55^ into the vector pTK-GLuc (NEB, N8084S). Primers were designed to amplify the RE1 using primerBLAST (http://www.ncbi.nlm.nih.gov/tools/primer-blast/). At the 5′-end of each primer additional nucleotides were introduced containing the restriction sites (capital letters) and additional nucleotides to facilitate digestion by the restriction endonucleases, a NotI restriction site 5′-tattGCGGCCGC-3′ was introduced into the forward primer 5′-TTTCTCTATCGATAGGTACAGGCA-3′ and a XhoI restriction site 5′-ctattCTCGAG-3′ was introduced into the reverse primer 5′-GTAATTCCACTTGTGACCAGGA-3′. PCR-cloning, digestion, ligation and sub-cloning were performed using standard molecular biology protocols. The pHRE-MP-GLuc HIF responsive construct has been previously described^56^. The pCMV-CLuc construct was from NEB (N0321S, pCMV-CLuc 2). Plasmid sequencing was performed by MWG Eurofins, Germany.

### Gaussia luciferase assay, transient and stable transfections

Gaussia luciferase assays were performed as previously described^56^. Briefly, at the selected time points, 10 μL of media was collected from the supernatant and stored at −20 °C. Gaussia luciferase activity was measured using the Biolux Gaussia luciferase Flex Assay kit (NEB) in a plate reader (Synergy HT, Biotek) and normalized to the luciferase activity of the secreted cypridina luciferase under the control of a constitutive CMV promoter (pCMV-CLuc) or protein concentration. Details and validation of the constructs are shown in Figure S4.

### Cell transfection with siRNA

Transient transfection with siControl (sc-37007, SCB) and siREST (s11932, Life Technologies) were performed as previously described^57^. Transfections with siRNA to be used in luciferase assays were performed in 24 well plates, as described above. All other experiments were performed on 6 well plates unless otherwise stated. In a typical experiment 200 K cells were seeded on 6 well plates and allowed to grow until approximately 60% confluent, at this time cells were transfected with 2 μL of Lipo, 100 μL Optimem and a pre-optimized amount of overexpressing construct (100 ng) or siRNA (100 pmol). On the day after transfection, cells were exposed to hypoxic or normoxic media. Transfection time with siRNA was kept constant for all experimental conditions. For RNA extraction experiments, siRNA was incubated for 48 hrs. For the preparation of whole cell protein extracts, siRNA was incubated for 72 hrs.

### Cycloheximide (CHX) pulse chase experiments

For the experiments where CHX-pulse chase was used to determine the protein stability of HIF-1α, REST and β-Actin in normoxia and hypoxia, 750,000 HEK293 cells where seeded on 6 cm dishes. The following day cells where conditioned to 24 hours of hypoxia or normoxia, after which cells where subjected to a pulse-chase treatment of cycloheximide (5 μg/mL) to block translation, and address the stability of the proteins in normoxia and hypoxia. Time points of CHX treatment after normoxic or hypoxic incubation included 0, 1, 2, 4, 8, 24 and 48 hours.

### qRT-PCR

#### cDNA was synthesized from

1 μg of RNA using MMLV (Promega), and amplified using the Prism 7900HT sequence detection system (Applied Biosystems, Foster City, CA) under default conditions. The mRNA relative expression was calculated by the ∆∆Ct method by normalizing the Ct of the samples to that of 18S rRNA (TaqMan Universal PCR Master Mix with the primer 18S rRNA-Euka, 4310893E, Life Technologies), followed by normalization to the control condition. The following REST qRT-PCR primers were used:

F: CGCCAGAGGGTGAAACTTTA; R: ATCCACAGCCATGAAGGAAG

### Western blot

All reagents were from Sigma unless otherwise stated. Standard protocols were used as previously described^57^,^58^. Mouse anti-β-actin, 1:10000, Sigma, A5441; Mouse anti-HIF-1α,1:1000, BD Pharmingen, 610958; Rabbit anti-REST, 1:1000, Abcam, ab28018; Rabbit anti-Lamin A/C, 1:1000, Cell Signalling, 2032; Mouse anti-α-Tubulin, 1:2000, SCB, sc-8035.

### ChIP assays

ChIP assays where performed as previously described^59^. The following ChIP qRT-PCR primers were used:

SYNJ1, F: TCCAGACACTCAGACTAGGAACTC, R: CCTGAAGAGCTGTCCATGGT, Probe: CCGTTTGCTGGGCTGTCGAC.The following antibodies were used:

Rabbit REST, 2 μg, Millipore, Rabbit IgG, 5 μg, Millipore, PP64B.

### Cell death using flow cytometry (YO-PRO and PI stains) and trypan blue exclusion assays

Trypan Blue (Sigma), was added 1:1 V/V to the cells, and live/dead cells where counted in an haemocytometer, by counting the Trypan blue permeabilised cells (dead), and the bright cells (alive), that where not Trypan Blue permeabilised. Data is represented as a percentage of the total population. Flow Cytometry staining to detect live and dead cells (early, late and end stage apoptotic cells) was performed using standard protocols. Briefly, cells where detached with Accutase solution, and stained with PI/YO-PRO, and counted in an Accuri C6 cytometer. Results are shown as the percentage of the total population under each condition.

### REST mRNA expression profiling

Human REST mRNA expression level according to anatomical distribution, or in different cell lines, was compiled from publically available microarray datasets using the Genevestigator software (https://genevestigator.com/gv/).

### RNA sequencing

HEK293 (100 thousand cells) were seeded on 6 well plates, transfected the next day with REST-RNAi or Ctrl-RNAi in 1 μL of Lipofectamine 2000. In the day after transfection cells were exposed to 1% oxygen using pre-conditioned media for 24 hours. RNA was isolated using QIAgen RNeasy columns as per supplier instructions. Total RNA electropherograms were used to evaluate RNA integrity, with integrity number for all samples being of the highest quality, RIN = 10, before being sent to the sequencing facility. Library construction began with 1 μg of total RNA. Since the protocol utilized was based on polyA capture, RNA was visualized on a BioRad Experion to insure RNA Quality Index values were greater than 8. For library construction the Illumina TruSeq v2 mRNA kit was utilized. This included also the poly(A)+ RNA isolation and DNase treatment. The protocol was followed according to manufacturer’s instructions except the final number of PCR cycles was 12 and not 15. Fifty base pare single end reads were prepared. Following construction, libraries were visualized by Bioanalyzer (Agilent) using the High Sensitivity Chip and quantified for pooling and sequencing using Kapa Biosystems qPCR quantitation kit according to manufacturer’s instructions. For sequencing libraries were diluted to 16 pM then applied to a V3 flowcell using the Illumina cBot according to manufacturer’s instructions. Sequencing was carried out on the Hi-Seq 2000 using HSCS v 1.5.15.1. Image analysis and base calling were carried out using RTA 1.13.48.0, and deconvolution and fastq file production were performed with CASAVA 1.8.2. Quality control metrics were obtained with Picard after Illumina FASTQ files alignment to the human genome hg19 reference sequence (GRCh37) using TopHat v2.0.8b^60^. Raw sequencing files and derived alignment files data are available in the ArrayExpress database (https://www.ebi.ac.uk/arrayexpress/) under the accession number E-MTAB-2580.

### RNA sequencing bioinformatics analysis

Illumina FASTQ files were aligned to the human genome hg19 reference sequence (GRCh37) using TopHat v2.0.8b^60^ (including Bowtie 2.1.0.0 and Samtools 0.1.19.0) with the -no-coverage-search option and the -G option to use a transcriptome index computed from the UCSC hg19 genes.gtf annotation. Transcript abundance and differential expression was estimated by direct comparison of normalized transcript counts from pairs of experimental conditions using the Cufflinks Cuffdiff2 program (v2.1.1)^61^ with default options and the UCSC hg19 genes.gtf annotation. To control for false positives, a second estimate of gene expression was computed using the HTSeq-count script (v0.6.1;^62^ followed by differential expression analysis with the Bioconductor edgeR package (v3.6.1)^63^, which uses a generalized linear model likelihood ratio test. Benjamini and Hochberg’s algorithm is used to control the false discovery rate (FDR) due to multiple testing^64^, those genes with FDR (q-value) < 0.05 were considered differentially expressed. The February 2009 human reference sequence (GRCh37) was produced by the Genome Reference Consortium (http://www.ncbi.nlm.nih.gov/projects/genome/assembly/grc/).

### Analysis of the promoter of REST-Dependent hypoxia repressed genes

Genomic sequences of genes identified as differentially regulated (“REST-Dependent genes repressed in hypoxia”) were extracted from the USCS Genome Browser database, selecting 2000 bases upstream (5′) of the transcriptional start site (TSS) and 1000 bases 3′ of the TSS. When multiple TSS sites were annotated for a single gene, the 3 Kb region was selected for all of them. The motif for the RE1 site from the JASPAR database (MA0138.2) was used as a frequency matrix and compared to the 3 Kb sequences by the pssm.search method using the Biopython Bio.motifs module, with a pseudocount of 0.5 and Normal background (equal frequency of G:A:T:C bases). The log odds threshold was set to 15, which would yield an expected false positive rate of approximately 0.05 against random background sequences. The sensitivity of this method was calibrated against equal sized sets of 3 Kb random sequences (using RSA-Tools) and against the target sequences with the RE1 motif bases shuffled (using EMBOSS).

We found 12 high stringency RE1 motifs (log odds score of 15) in the promoter region (−2 Kb/+1 Kb) of REST-dependent genes repressed in hypoxia (Fig. 6B). This number of hits is much higher than what could be found in control experiments, when the RE1 motif was searched against an equal sized set of random sequences (2.0034 ± 0.54 gene hits, Fig. 6C) or randomly shuffling the promoter sequences from the REST genes (0.4 ± 0.54 gene hits, Fig. 6C). This analysis strengthen the point that the REST-dependent genes repressed in hypoxia have consensus RE1 motifs in the −2 Kb/+1 Kb region of their promoter in a greater frequency than one would expect by chance.

### Functional analysis of gene datasets using PANTHER and DAVID

PANTHER was used for the functional annotation of gene lists (Figures S1A–J) and for the statistical overrepresentation tests (Fig. 1C–H), significance threshold was set as p < 0.05 for the binomial test^65^,^66^. This is a hypergeometric test which first assigns each gene to one or more GO or PANTHER categories, and then tests if there are more genes in a particular category than those one would expect by random chance in a dataset of similar size. DAVID Functional Annotation Clustering was performed with default settings, the significance threshold of this gene-enrichment analysis was set as p < 0.05 for the EASE Score (a modified Fisher Exact p-Value)^67^,^68^.

### Statistical analysis

All experiments were performed at least 3 independent times. Data is shown as mean ± SEM. Statistical significance was tested in Prism (Graphpad), using Student’s t test for the comparison of two data sets or ANOVA for more than two datasets. *p < 0.05, **p < 0.01 and ***p < 0.001.

## Additional Information

**How to cite this article**: Cavadas, M. A. S. *et al*. REST is a hypoxia-responsive transcriptional repressor. *Sci. Rep.* **6**, 31355; doi: 10.1038/srep31355 (2016).

## Supplementary Material

Supplementary Information

Supplementary Figure 2

Supplementary Table 1

Supplementary Table 2

Supplementary Table 3

Supplementary Table 4

Supplementary Table 5
