# Supplementary material for: REST is a hypoxia-responsive transcriptional repressor
Source: Sci Rep. 2016 Aug 17;6:31355. doi: 10.1038/srep31355 (PMC4987654; doi:10.1038/srep31355)
Supplement: Supplementary Information [file srep31355-s1.pdf]

SUPPLEMENTARY INFORMATION

REST is a hypoxia-responsive transcriptional repressor.

Miguel A. S. Cavadas[1,2,3], Marion Mesnieres[2], Bianca Crifo[2], Mario C. Manresa[2], Andrew C. Selfridge[2], Ciara E Keogh[2], Zsolt Fabian[2], Carsten C. Scholz[1,2,10], Karen A. Nolan[4,10], Liliane M. A. Rocha[5], Murtaza M. Tambuwala[6], Stuart Brown[7], Anita Wdowicz[8], Danielle Corbett[8], Keith J. Murphy[8], Catherine Godson[4], Eoin P. Cummins[2], Cormac T. Taylor[1,2,*] and Alex Cheong[1,2,9,*]

[1] Systems Biology Ireland, University College Dublin, Dublin 4, Ireland.

[2] Conway Institute of Biomolecular and Biomedical Research, School of Medicine and Medical Sciences, University College Dublin, Dublin 4, Ireland.

[3] Instituto Gulbenkian de Ciência, Rua da Quinta Grande, 2780-156 Oeiras, Portugal.

[4] Diabetes Complications Research Centre, School of Medicine and Medical Sciences, University College Dublin, Dublin 4, Ireland.

[5] Faculdade de Medicina, Universidade de Lisboa, 1649-028 Lisbon, Portugal.

[6] School of Pharmacy and Pharmaceutical Sciences, University of Ulster, Coleraine, Co. Londonderry, BT52 1SA, Northern Ireland, UK.

[7] Center for Health Informatics and Bioinformatics, New York University School of Medicine, New York, NY 10016, USA.

[8] Neurotherapeutics Research Group, UCD School of Biomolecular and Biomedical Science, Conway Institute, University College Dublin, Belfield, Dublin 4, Ireland.

[9] Life and Health Sciences, Aston University, Birmingham , B4 7ET, UK.

[10] Institute of Physiology and Zurich Centre for Integrative Human Physiology, University of Zurich, Zurich, Switzerland.

*These authors contributed equally to this work

Correspondence: Dr Alex Cheong, Life and Health Science, Aston University, Birmingham B4 7ET, +44-1212043420, email: a.cheong@aston.ac.uk

Supplementary Figures

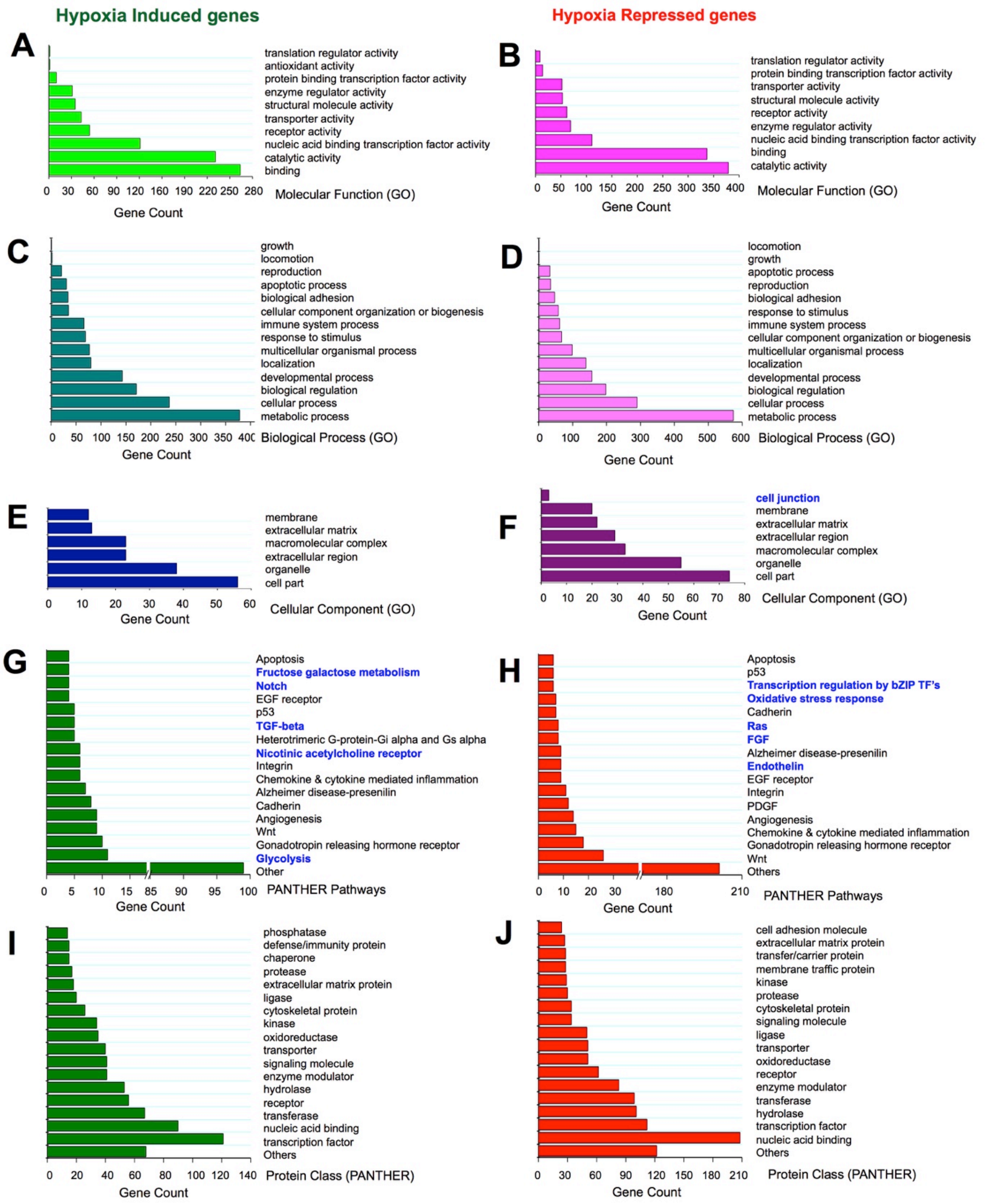

Figure S1. Ontological and overrepresentation analysis of the differentially expressed genes in hypoxia. (**A-J**) PANTHER was used to classify the genes involved in each subset of hypoxia induced and repressed genes according to GO terms, and PANTHER protein class and pathway terms. Highlighted in blue are the terms whose gene count was present in the top 20 of only one of the lists (induced or repressed).

Supplementary Figure S2 is in its separate file.

Figure S2. REST mRNA expression across multiple cell lines. REST mRNA expression compiled from publically available microarray datasets of 1562 different cell lines. The graph was compiled with Genevestigator and shows the human REST mRNA expression levels (xx axis) and number of samples (yy).

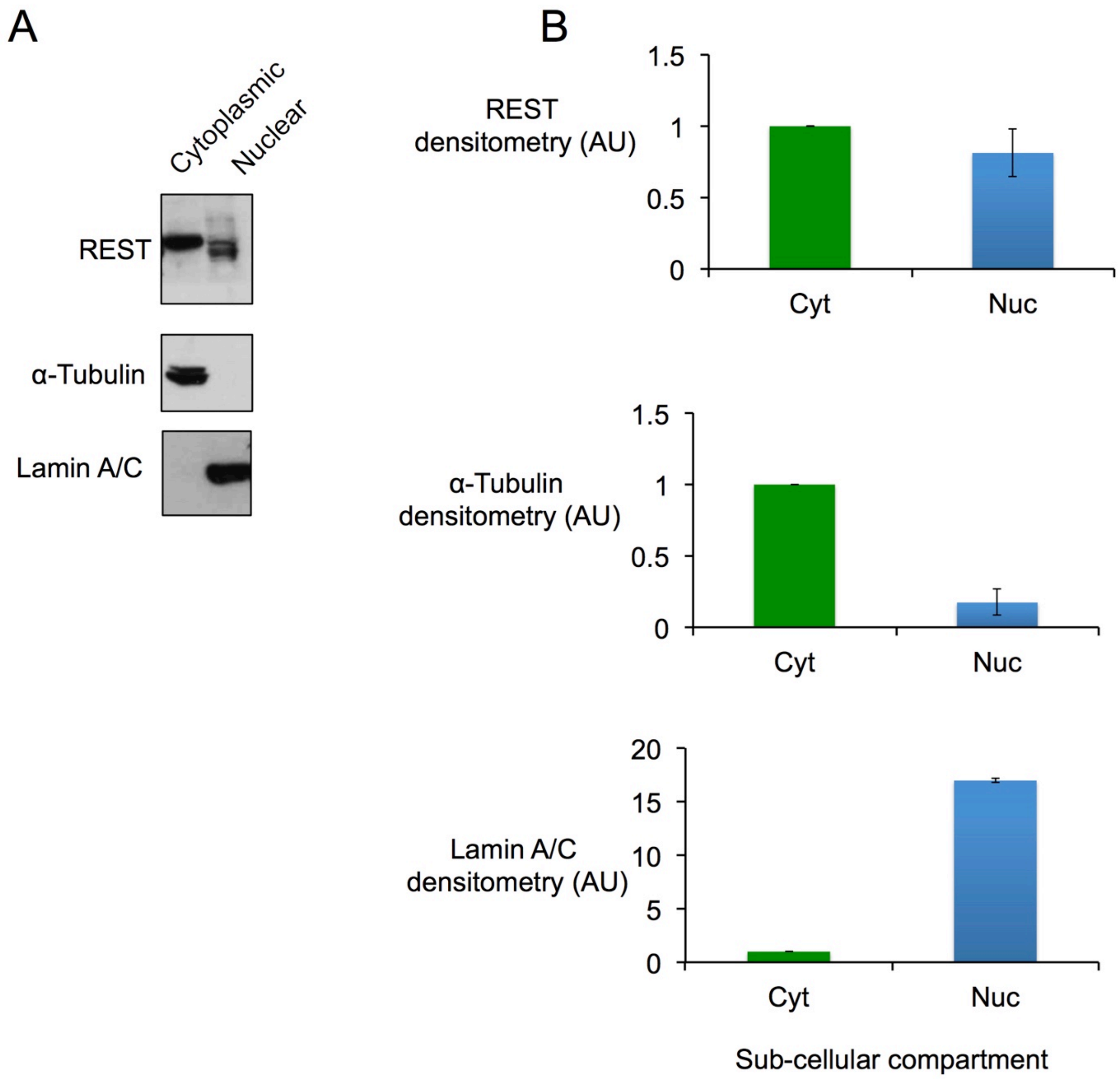

Figure S3. REST is expressed in the nucleus and cytoplasmic of normoxic HEK293 cells. (A) Nuclear and cytoplasmic extracts of HEK293 cells where prepared and immunobloted for REST, Lamin A/C (nuclear loading) and α-Tubulin (cytoplasmic loading). 20 to 40 ug of total lysates where loaded per experiment, in a total of 8 independent experiments. **(B)** Densitometry of (A), for REST, Lamin A/C and α-Tubulin normalized to the cytoplasmic expression level. Data are represented as mean ± SEM, *n* = 8 independent experiments.

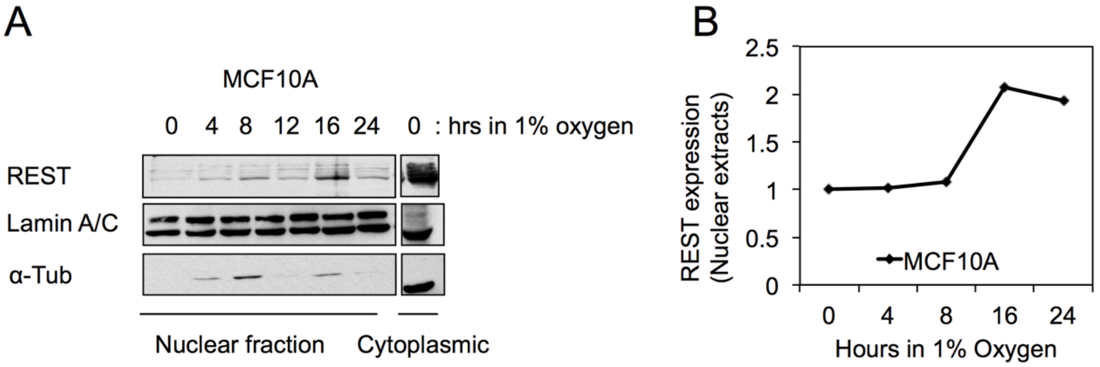

Figure S4. REST accumulates in the nucleus of hypoxic MCF10A cells. (A) Cells where exposed to hypoxia (1% oxygen) for the indicated time points. Nuclear and cytoplasmic extracts of MCF10A cells where prepared and immunobloted for REST, Lamin (nuclear loading) and α-Tubulin (cytoplasmic loading). **(B)** Densitometry of (A). Data are represented as mean ± SEM, *n* = 3 independent experiments.

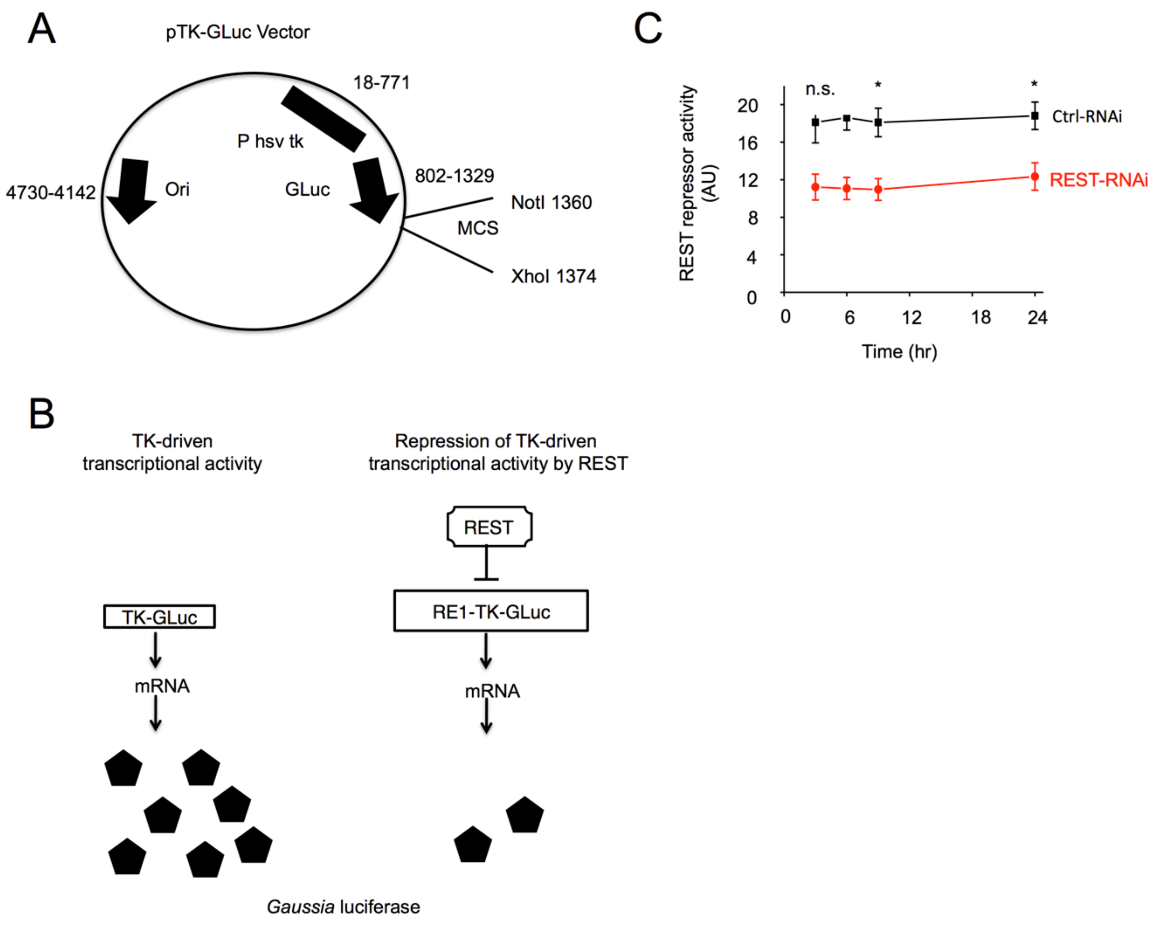

Figure S5. Development and validation of a RE1 responsive *Gaussia* luciferase promoter reporter construct (pRE1-TK-GLuc). (A) The pTK-GLuc Vector is a mammalian expression vector that encodes the secreted luciferase from the copepod *Gaussia princeps* as a reporter, under the control of the constitutive Herpes Simplex Virus thymidine kinase promoter (P hsv tk)). *Gaussia* Luciferase (GLuc) is a 20 kDa protein encoded by a "humanized" sequence, and it contains a native signal peptide at the N-terminus that allows it to be secreted from mammalian cells into the cell culture medium (Tannous et al., 2005; Verhaegen and Christopoulos, 2002). pTK- GLuc has a multiple cloning site (MCS) downstream of the GLuc stop codon, XhoI and NotI were chosen as entry sites for the RE1 sequence. Ori indicates the bacterial replication origin of replication (pMB1), for the propagation and maintenance (sub cloning) in *E. coli.* (https://www.neb.com/products/n8084-ptk-gluc-vector). **(B)** The pRE1-TK-GLuc REST responsive construct was developed by incorporating the RE1 originally found in the *SCN2A* gene (Mori et al., 1992) into the vector

pTK-GLuc. The pTK-GLuc reporter construct, constitutively expresses *Gaussia* luciferase under the control of the constitutive Herpes Simplex Virus thymidine kinase promoter, incorporation of the RE1 site is aimed at assessing how strongly REST binds and inhibits transcription. **(C)** HEK293 cells were transfected with a vector expressing Gaussia luciferase under the control of the TK promoter (pTK-GLuc) for constitutive expression, or with the REST responsive pRE1-TK-GLuc construct. Results are shown as REST repressor activity (dividing the RLU of pTK- GLuc by pRE1-TK-GLuc). REST knockdown was performed using REST specific (REST-RNAi) or scrambled control RNAi. Data are represented as mean ± SEM, n = 3 independent experiments. * $p<0.05$. AU = Arbitrary Units.

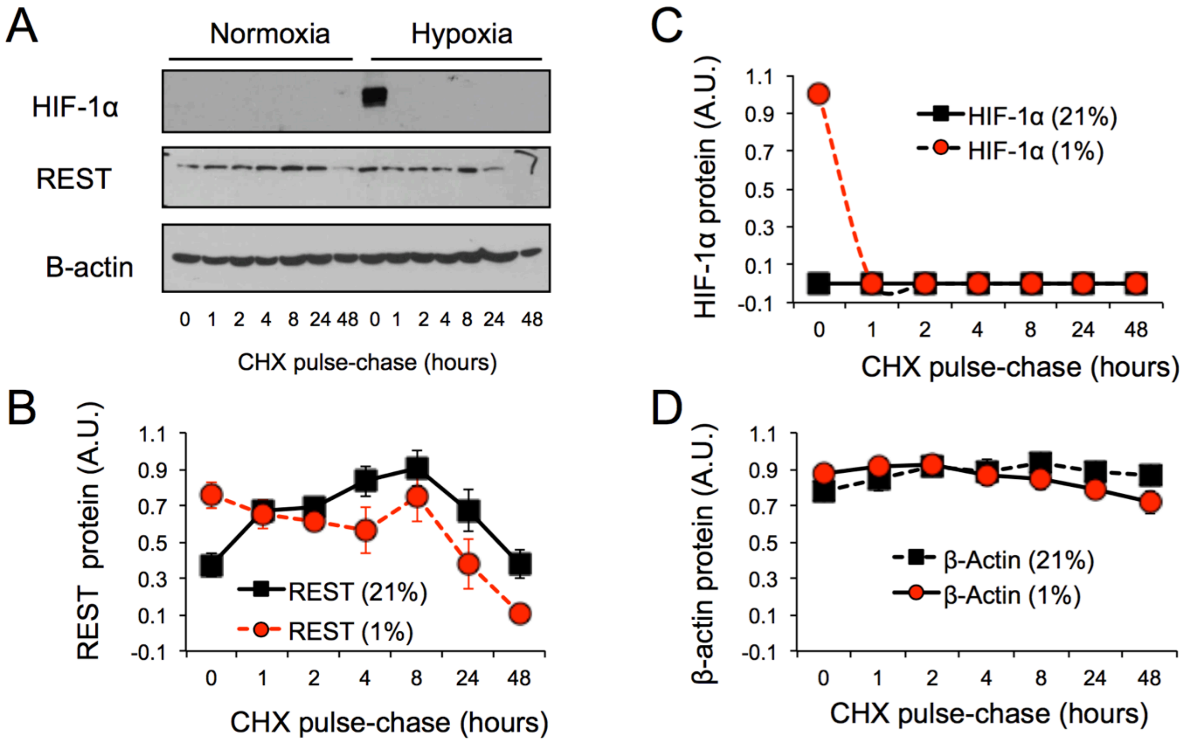

Figure S6. REST protein stability is not affected by hypoxia. (**A**) HEK293 cells were pre-conditioned to hypoxia or normoxia for 24 hours and subjected to cycloheximide (CHX) pulse-chase treatments for the indicated time points before whole cell extracts were prepared and immunoblotted as indicated for HIF-1α, REST and β-actin (n = 4). Densitometric analysis of (**A**) for REST **(B),** HIF-1α **(C)** and β-actin **(D)** are presented.

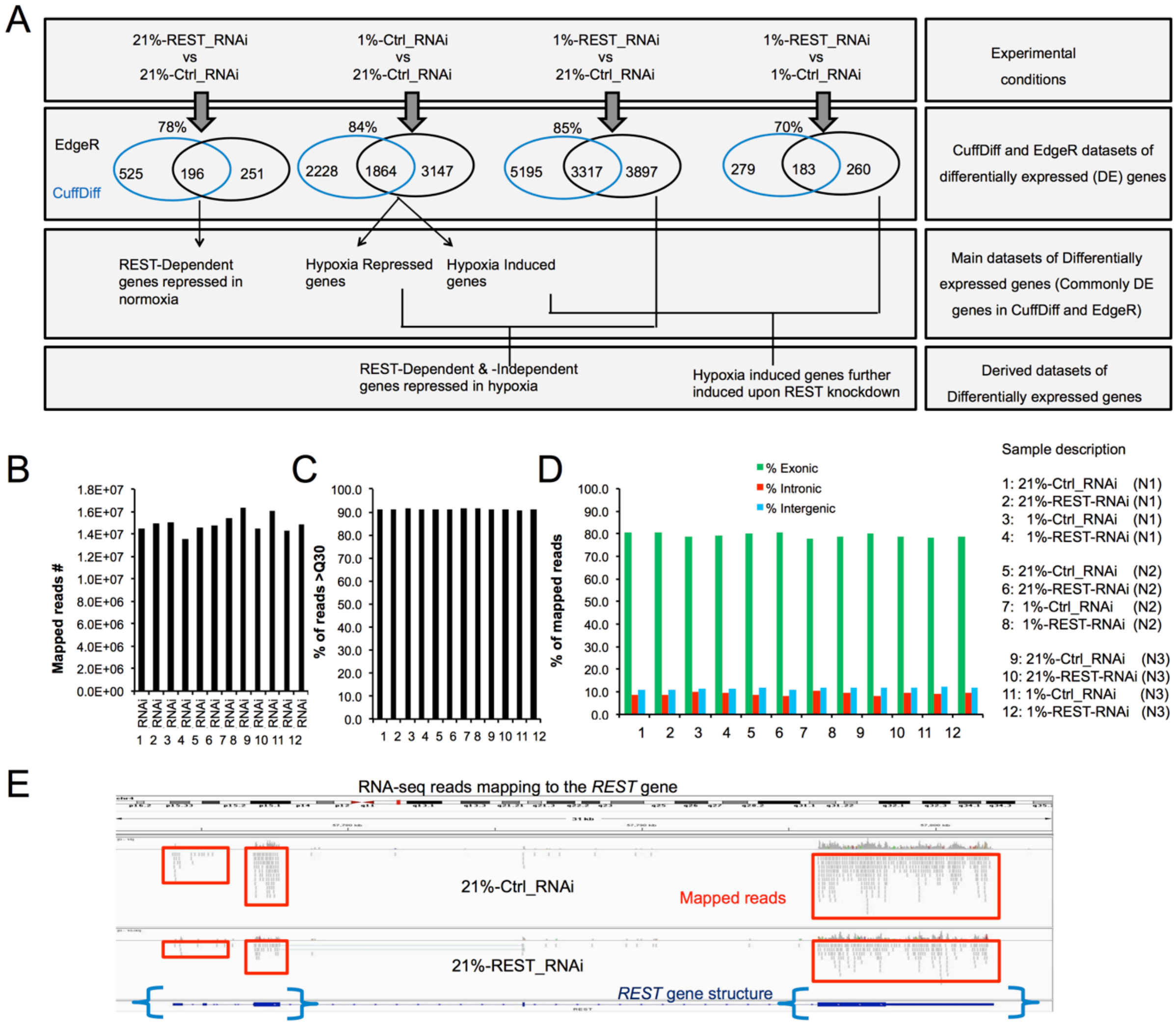

Figure S7. Technical validation of RNA-seq and RNAi mediated knockdown experiments. (**A**) EdgeR and Cuffdiff were used to independently estimate the magnitude and significance of the gene expression changes, with an agreement between 70 to 85%. Commonly differentially expressed genes (q-value<0.05) were selected for further analysis. REST-Dependent genes repressed in normoxia (21% O_2) were defined as those genes whose expression was increased in normoxic cells treated with REST-RNAi as compared to Ctrl-RNAi treated cells. Hypoxia-Repressed and -Induced genes were those genes whose expression decreased or increased, respectively, in hypoxic cells (1% O_2) as compared to normoxic cells. REST-Dependent genes repressed in hypoxia were those genes whose expression was decreased in hypoxic cells, but not when hypoxic cells were treated with REST-RNAi. REST-Dependent hypoxia induced genes were defined as those genes that were

induced by hypoxia, and further induced by REST-RNAi treatment in hypoxia. (**B**) Around 15 million mapped reads were obtained consistently between the 12 different samples, independent of treatment and biological replicate. (**C**) Quality of the reads as assessed by Q30 parameter which indicates an accuracy of 99.9 % of base calling, more than 90% of the mapped reads had an QC30. (**D**) The percentage of reads mapping to intronic, exonic and intergenic regions is shown for each RNA-seq sample. The high percentage of exonic reads in our poly(A)+ RNA samples indicates good enrichment of poly(A)+ RNA and low DNA contamination. Furthermore, it indicates that our sequencing matches well with known exon annotation. (**E**) Alignment of reads by TopHat to human reference genome hg19 (GRCh37) from sample 1 (21%-Ctrl_RNAi) and sample 2 (21%-REST_RNAi) to the *REST* gene on chromosome 4q11. Visualized with IGV 2.3.32. RNA-seq reads mapping to the REST gene show a preferential allocation to exons, indicating poly(A)+ RNA enrichment, and a marked decreased in the number of reads upon REST-RNAi treatment indicating the validity of RNA-seq to accurately measure differences in gene expression.

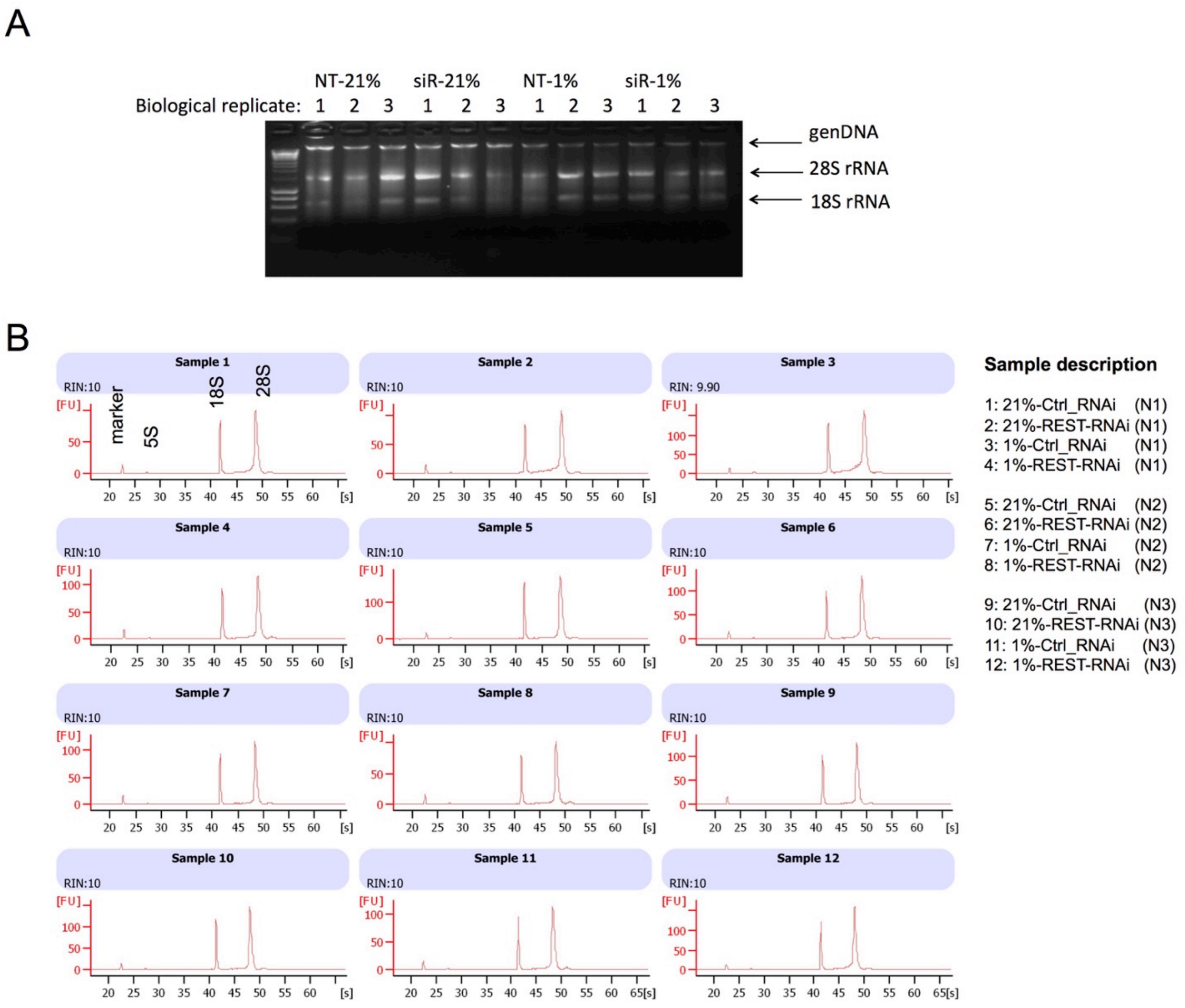

Figure S8. Agarose gel and Bioanalyzer electropherogram of total RNA used in the RNA-Seq experiment. (A) 2 μg of RNA from each experimental conditions and biological replicate was loaded into a 2% agarose gel with ethydium bromide and run on TAE buffer. 18S and 28S indicate ribosomal RNA subunits. gDNA, indicates genomic DNA contamination. **(B)** Around 100 ng of RNA were run on Eukaryote Total RNA Nano bioanalyzer from Agilent technologies. Distinct ribosomal peaks corresponding to 18S and 28S eukaryotic RNA and a relatively flat baseline between the 5S and 18S ribosomal peaks can be seen for all samples, as well as RIN=10 in all samples. Marker indicates the peak from an internal control molecular weight marker. N indicates the biological replicate, 21% and 1% indicate the oxygen percentage, Ctrl-RNAi and REST-RNAi indicate scramble control and REST targeting siRNA, respectively.

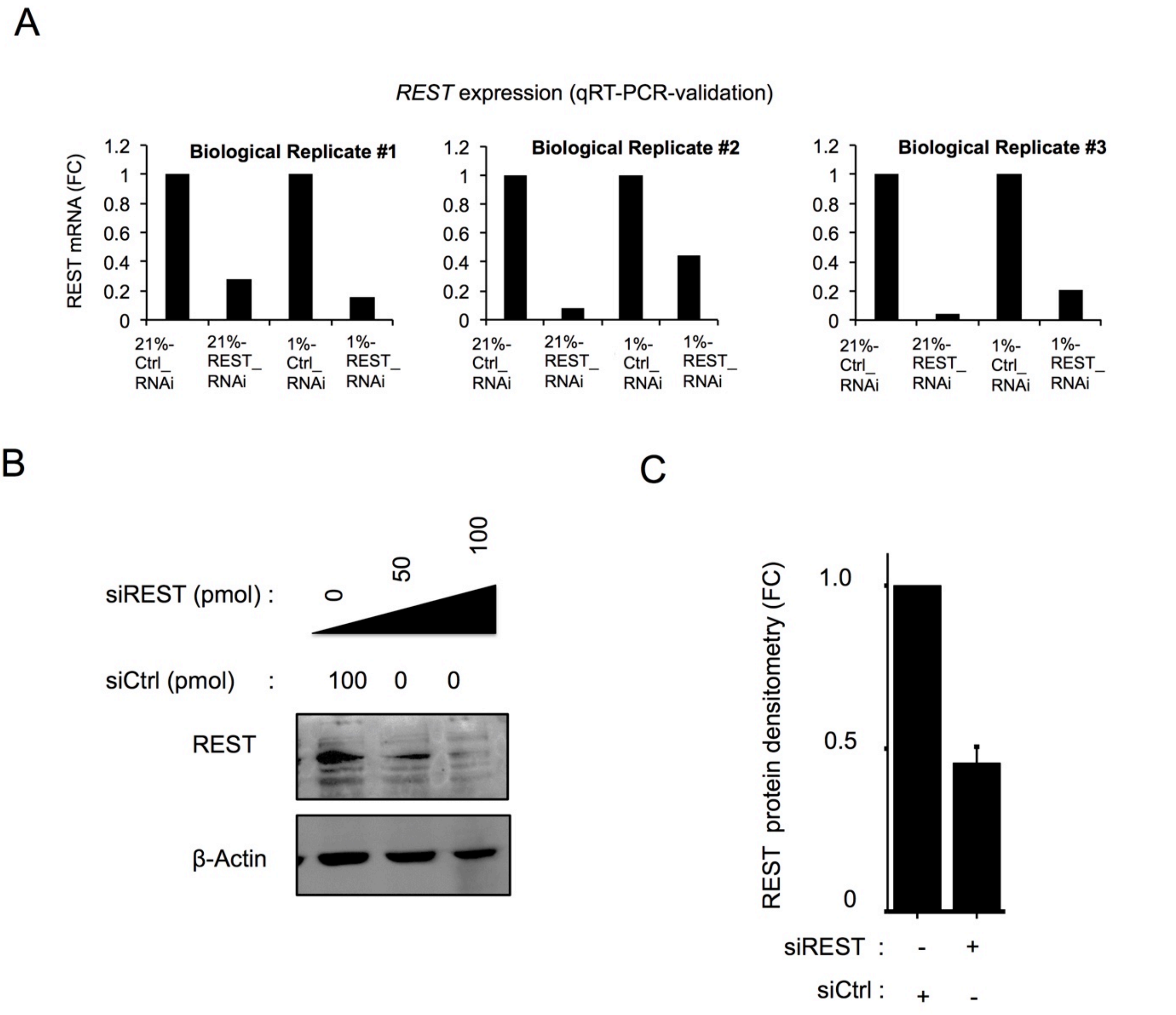

Figure S9. Validation of REST mRNA knockdown by qPCR, on the mRNA samples used for RNA-seq and by western blot. (A) HE293 mRNA used for RNA-seq analysis where exposed to normoxia (21 % oxygen), hypoxia (1 % Oxygen) with Control-RNAi or REST-RNAi. Each graph shows one biological replicate. Data is normalized to control RNAi in normoxia or hypoxia. **(B)** HEK293 cells where treated as described in (A), in normoxia, and immunobloted with the indicated antibodies. **(C)** Densitometry of 3 independent experiments using the optimized REST siRNA quantity: 100 pmol.

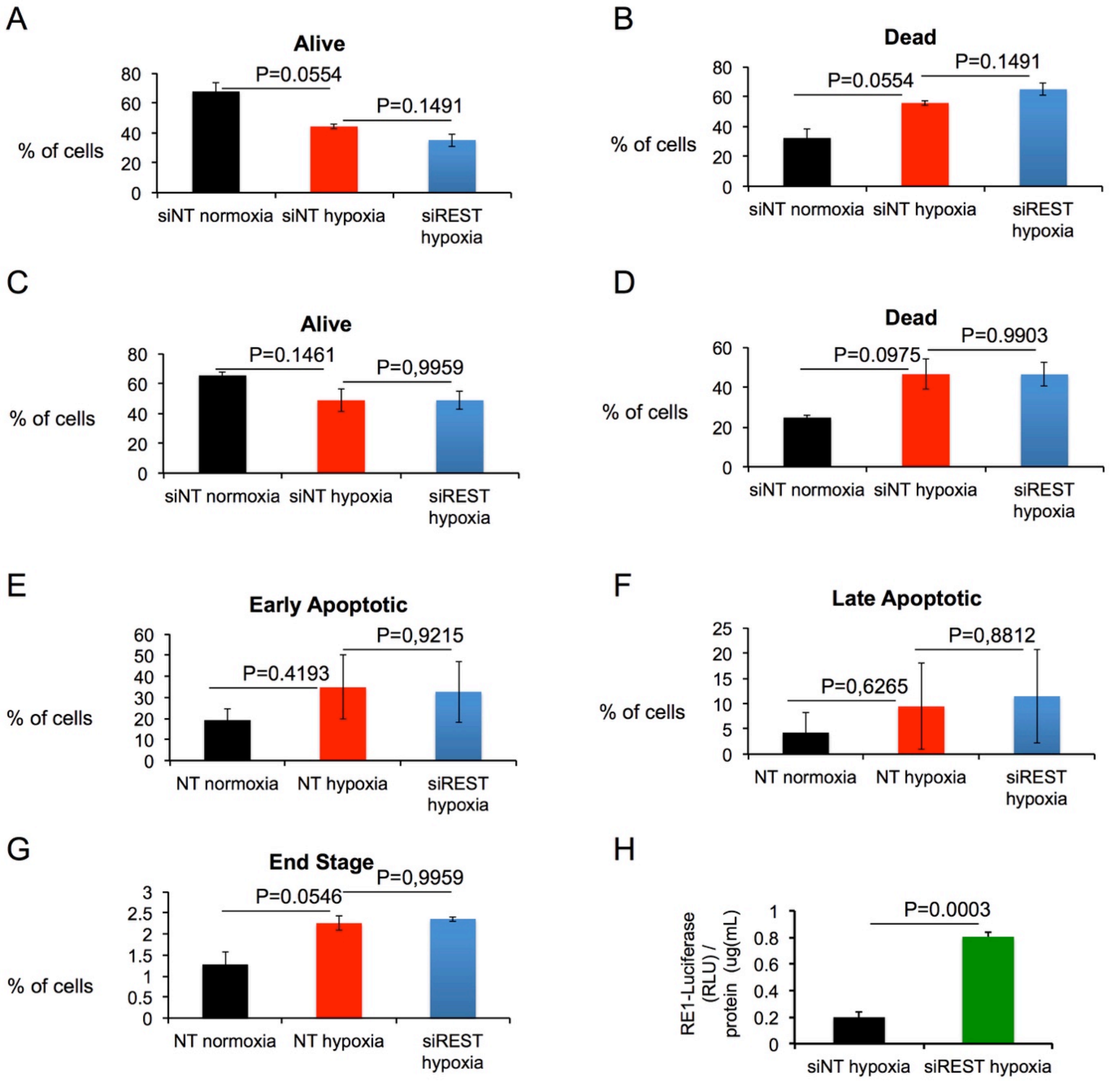

Figure S10. Analysis of cell death under hypoxia during REST knockdown using siRNA. HEK293 cells where treated with non targeting control siRNA (siNT) or REST siRNA, exposed to normoxia or hypoxia (1% oxygen) for 48 hours. **(A, B)** Cells where collected and analysed for cell viability using the Trypan Blue exclusion assay, total alive **(A)** or dead cells are presented **(B)** as a percentage of the total population counted for each treatment. **(C-H)** Cells where treated as in **(A, B)** and analysed by Flow Cytometry with the PI/YO-PRO stains. Total alive **(C)** and dead **(D)** cells are shown, as well as Early apoptotic **(E)**, late apoptotic **(F)** and end-stage apoptotic cells **(G)**. **(H)**. HEK293 cells where treated with non-targeting control siRNA (siNT) or REST siRNA, and with the RE1-Gaussia luciferase reporter. Cells were then exposed to hypoxia for 24 hours, and the activity of the reporter was measured. Successful REST knockdown is confirmed by the increased luciferase

activity of the RE1-reporter, which is repressed in the presence of REST. Data are represented as mean ± SEM, n = 3 independent experiments. P-values are shown, $p<0,05$ is considered significant.

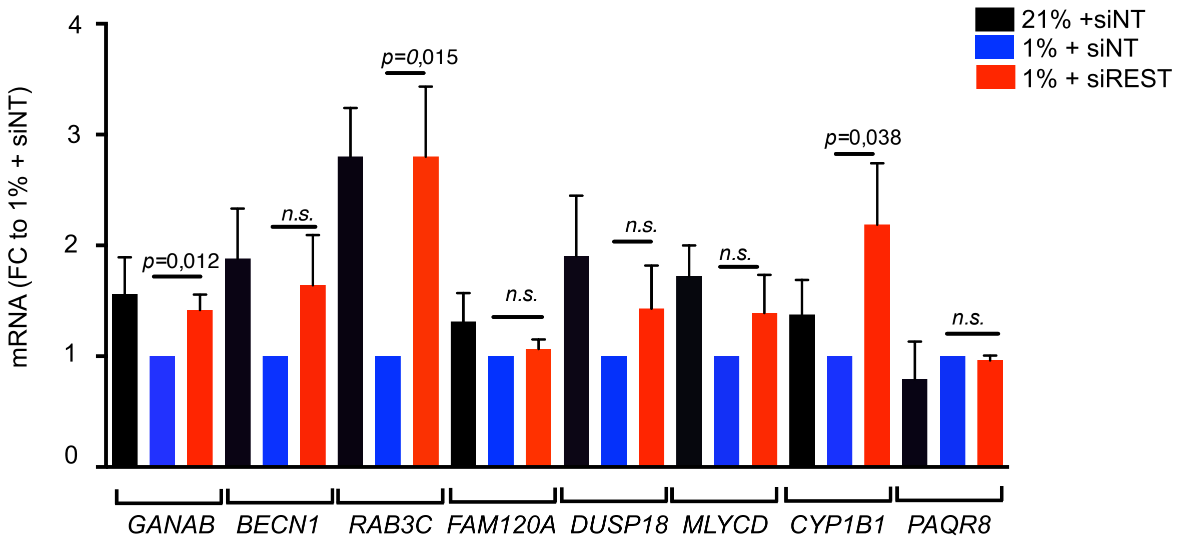

Figure S11. Validation of REST dependent hypoxia repressed genes. qRT-PCR was used to validate the mRNA expression of the indicated REST target genes. mRNA was isolated from HEK293 cells treated for 24 hours to normoxia (21% oxygen) or hypoxia (1% oxygen), in the presence or absence of an siRNA targeting REST. Data are represented as mean ± SEM, n = 4 independent experiments. P-values are shown, p<0,05 is considered significant over 1% oxygen siNT treated samples.
